# Integration of a smart multidose blister package for medication intake: A mixed method ethnographic informed study of older adults with chronic diseases

**DOI:** 10.1371/journal.pone.0262012

**Published:** 2022-01-21

**Authors:** Sadaf Faisal, Jessica Ivo, Ryan Tennant, Kelsey-Ann Prior, Kelly Grindrod, Colleen McMillan, Tejal Patel

**Affiliations:** 1 School of Pharmacy, University of Waterloo, Kitchener, Ontario, Canada; 2 Faculty of Engineering, Systems Design Engineering, University of Waterloo, Waterloo, Ontario, Canada; 3 Centre for Family Medicine Family Health Team, Kitchener, Ontario, Canada; 4 Renison University College, University of Waterloo, Waterloo, Ontario, Canada; 5 Schlegel–University of Waterloo Research Institute of Aging, Waterloo, Ontario, Canada; University of Manitoba, CANADA

## Abstract

Smart adherence products are marketed to assist with medication management. However, little is known about their in-home integration by older adults. It is necessary to investigate the facilitators and barriers older adults face when integrating these products into their medication taking routines before effectiveness can be examined. The aim of this study was to (a) examine the integration of a smart multidose blister package and (b) understand medication intake behaviour of adults with chronic diseases using an integrated theoretical model comprised of the Technology Acceptance Model (TAM), Theory of Planned Behaviour (TPB) and Capacity, Opportunity, Motivation and Behaviour (COM-B) Model. An ethnographic-informed study was conducted with older adults using the smart multidose blister package to manage their medications for eight weeks. Data was collected quantitatively and qualitatively using in-home observations, photo-elicitation, field notes, semi-structured interviews, system usability scale (SUS) and net promoter scale (NPS). The interview guide was developed with constructs from the TAM, TPB and COM-B Model. Data were analyzed using the Qualitative Analysis Guide of Leuven (QUAGOL) framework to generate themes and sub-themes which were mapped back to TAM, TBP and COM-B Model. Ten older adults with an average age of 76 years, of which 80% were female, participated in the study. On average, participants reported five medical conditions, while the average number of medications was 11.1. The mean SUS was 75.50 and overall NPS score was 0. Qualitative analysis identified three themes; (1) factors influencing medication intake behaviour (2) facilitators to the product use and, (3) barriers to the product use. The smart blister package was found to be easy to use and acceptable by older adults. Clinicians should assess an older adult’s medication intake behavior as well as barriers and facilitators to product use prior to recommending an adherence product for managing medications.

## Introduction

The National Council of Aging, United States of America (USA), reported that 80% of older adults are diagnosed with one chronic medical condition, and 77% have at least two or more chronic medical conditions [1]. A cross-sectional study of the prevalence of multimorbidity highlighted that the number of multiple chronic diseases is directly proportional to age; 64.9% of people aged 65–84 reported two or more, and 81.5% of people aged >85 years reported more than three chronic diseases [2]. The usage of medications increases with the number of chronic diseases a person has. A Canadian study found that the usage of more than five medications increased from 17.8% to 63.8% in patients with the presence of three or more medical conditions [3]. Another USA family residency practice study revealed that 86.1% of patients diagnosed with more than two chronic medical conditions received five or more prescription orders at one office visit [4]. Chronic diseases generally require long term use of medication therapies with multiple medications, especially if multiple chronic conditions exist [5]. Administering multiple medications on a regular basis is a challenging task for older adults with chronic diseases due to increased symptom burden, complex medication regimens, physical and cognitive deficits, and adverse effects leading to treatment non-adherence [6].

Appropriate medication adherence has been linked to improving health outcomes, quality of life and reducing healthcare system costs in patients with chronic diseases [7–9]. Despite this evidence, non-adherence to therapies is still considered one of the major issues that healthcare systems face globally. Approximately half of patients with chronic diseases in developed countries do not adhere to their medications [10]. A recent study examining the prevalence of medication non-adherence in patients with chronic disease in the USA demonstrated that improving adherence to antihypertensive medications could result in 117,594 fewer emergency room visits and over 7 million fewer inpatient hospital stays annually [11]. The study also reported that adherence to antidiabetic, antihyperlipidemic and antihypertensive therapies can lead to a healthcare cost saving of $ 4.5 billion, $5 billion and $14 billion per year, respectively [11].

Medication non-adherence is a multidimensional process, and several factors play an essential role when it comes to adherence. A systematic review found 771 determinants of non-adherence based on five factors [12]. These factors are related to patient (attitude, belief and knowledge about medications, forgetfulness), therapy (complex regimen, previous treatment failure, side effects, medication cost), disease (severity of symptoms), healthcare systems (patient-provider relationship, access to treatment resources) and socio-economic determinants (illiteracy, unemployment, social support network) [10, 12]. Although non-adherence is not directly correlated with age, its prevalence and risks are reported to be higher in older adults due to a combination of factors, including multimorbidity, cognitive impairment, polypharmacy, drug-related adverse effects and drug storage or formulation issues [12, 13]. Understanding factors influencing a person’s ability to take their medications appropriately is vital to identify patients at risk of non-adherence, assess the reasons for non-adherence and provide individualized adherence interventions.

People with chronic diseases often perform common behaviours to manage multiple medications with complex regimens across a continuum of care [14, 15]. To name a few, these behaviours may include preparing, administering and procuring medications, managing side effects, and communicating with healthcare providers [15]. These behaviours may ultimately impact adherence and as such, it is important to explore facilitators and barriers which influence these behaviours. Health behaviour theories play an important role in understanding why people do or do not practice health related behaviours, identifying a wide range of factors that can impact patient’s medication intake, and designing patient specific interventions to improve medication intake [16–18].

In the last two decades, the introduction of telehealth technologies has reformed the utilization of and access to healthcare systems and resources. Specifically, to address non-adherence and provide support for in-home medication intake, there has been an increasing development of smart technology-based products. These products range from mobile phone applications, electronic reminders via mobile phone text messages or emails to smart medication dispensing products that offer real-time medication intake monitoring via web or cloud-based portals [19–22]. A recent review identified 51 smart medication adherence products, of which 38 were commercially available for in-home patient use [23]. Most of these products were marketed by their manufacturers as user-friendly; however, not all of these products were tested with real-world in-home patient use. Another scoping review identified ten studies that evaluated the integration of prototype and commercially available smart oral multidose dispensing systems and reported them to be usable by patients [24]. However, one of the gaps identified by this scoping review was that despite having the capacity to dispense multiple medications, only two studies used a product for more than once daily administration of multiple medication administration [24]. Smart technology-based adherence products may have great potential for supporting patients with their medication management as well as allowing healthcare providers to monitor patients on a real-time basis, however, it is imperative to understand the barriers and facilitators of integrating these products into patients’ homes to achieve their full benefits. Additionally, in order for these technologies to be effective they must be accepted by end users [25]. Different technology adoption models have been identified in the literature to investigate the usability and acceptance of technology-based systems or products [26]. These theoretical frameworks can help explain the attributes affecting the acceptance or refusal of a technological intervention.

Therefore, we designed a mixed method ethnographic informed study to examine the integration of a prototype smart multidose blister package for in-home patient use to manage complex therapy regimens and to explore their medication intake behaviour by using the Technology Acceptance Model (TAM), Theory of Planned Behaviour (TPB), and Capability, Opportunity, Motivation-Behaviour (COM-B) Model.

### Ethical consideration

This study received ethics approval from the University of Waterloo Clinical Research Ethics Committee. All participants provided written informed consent and had the right to withdraw at any stage of the study.

## Materials and methods

### Theoretical frameworks

#### Technology acceptance model

The TAM provides a framework to understand a person’s intention to use a product versus their actual use. TAM describes that the use of technology depends on a person’s perception of ease of use and usefulness of the technology along with external factors such as system characteristics, user training and implementation [27]. TAM has been used in the healthcare research field to understand technology acceptance in older adults [28, 29].

#### Theory of Planned Behaviour

The TPB provides a theoretical framework to understand variables that can affect behaviour change [30]. This theory explains that a person’s behaviour is constructed on their intention to perform the behaviour. The intention to engage in a behaviour can be driven by an individual’s positive and negative estimations about the behaviour, how other people in life approve or disapprove of the behaviour and the beliefs about the resources available or skills needed to perform the behaviour [31]. Various adherence studies have used TPB to identify determinants of non-adherence and improve treatment adherence [32, 33].

#### Capability, Opportunity, Motivation-Behaviour (COM-B) Model

The COM-B Model is a comprehensive behaviour system that provides structure to assess different factors affecting the implementation of behaviour change [34]. This model explains that for an individual to be motivated for a behaviour such as medication intake, they must have sufficient capability and opportunity. In addition, various social and environmental factors, (e.g. lack of healthcare resources, access to the medications, cost, and social support for medication management) can influence consistent medication intake.

### Smart multidose blister package

**Fig 1:**
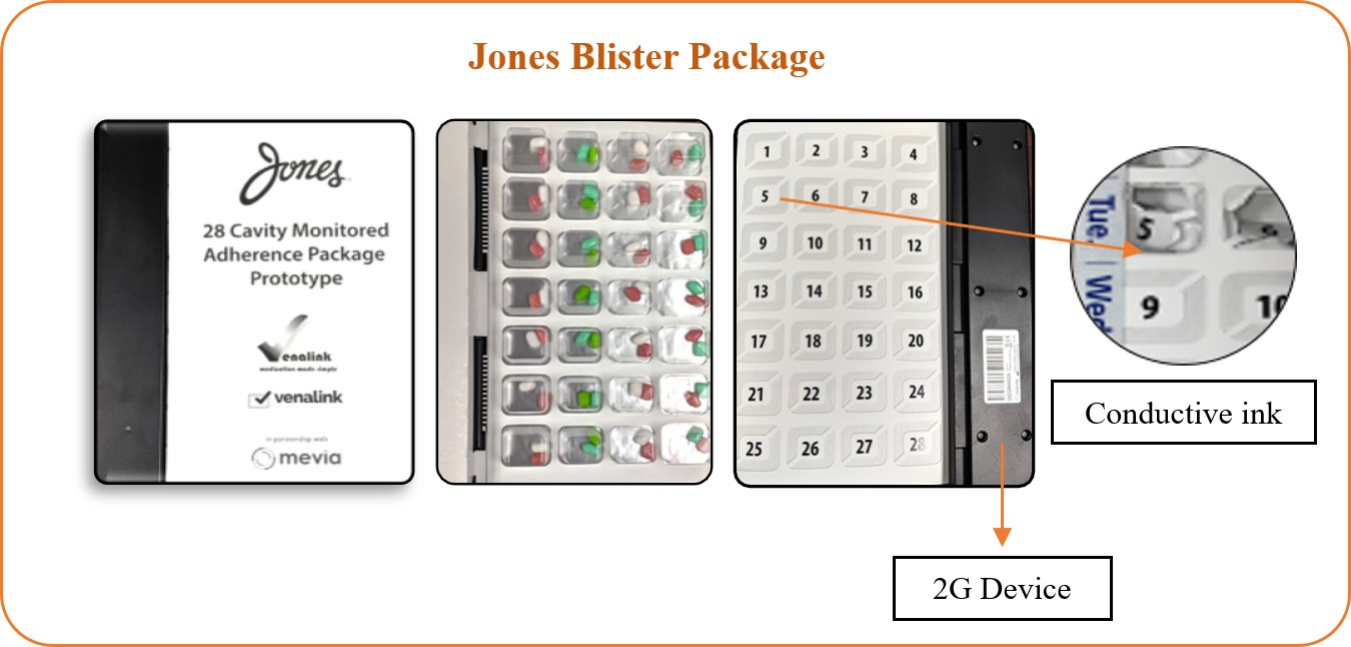
Smart multidose blister package.

The smart multidose blister package is a prototype product with telecommunications technology (see Fig 1).

**Fig 2:**
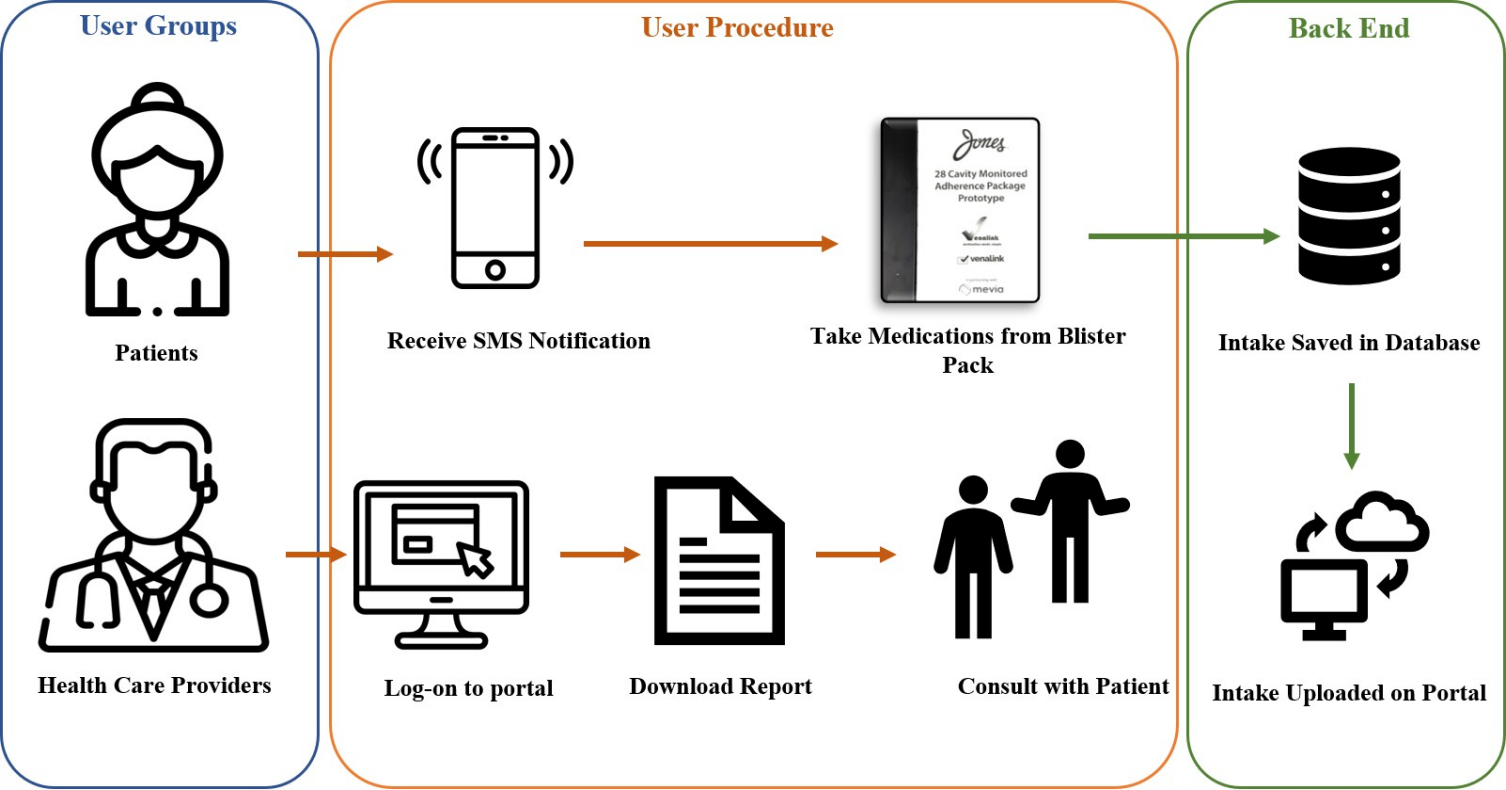
Utilizing smart multidose blister package.

The blister package consists of a plastic blister, aluminum foil substrate, and paperboard with conductive ink circuitry that enables the recording of dosage events. The blister package is comprised of 28 cavities and provides up to four times dosing of multiple medications for one week. A telecommunications device is attached to the individual disposable blister pack. The package is pre-filled by the pharmacy. When the cavity is broken to access the medications, the circuitry ink linkage breaks and the telecommunications device records the medication intake event and uploads the data to a cloud-based software portal. The system generates text reminders and notifications via a global system for mobile communications (GSM) and short message service (SMS) technology, to a mobile phone or email address. The software portal is an online interface that can be accessed by a healthcare professional (pharmacist or clinician) or a caregiver. The portal can be used to set patient medication schedules, set up notifications, and obtain a report on patient medication adherence (see Fig 2). The portal displays all information transmitted by the telecommunications device and includes a summary page displaying events for all patients/individuals attached to a user’s account. Additionally, each account has a patient profile page providing patient information, device identifier, battery status, service connection status, and date range for monitoring.

### Study design

A mixed method ethnographic informed study design was used to examine the integration of the smart multidose blister package and gain an in-depth understanding of the processes, activities and behaviours around medication intake in patients with chronic diseases. Ethnography is a qualitative research method that involves learning about a culture-sharing group of people by being immersed in their natural environment [35, 36]. People with chronic diseases are often on multiple medications and have complex medication regimens, which was considered a culture-sharing aspect in this study. For the purpose of this study, we defined integration as the use of the product to support daily medication intake.

### Study participants

We used a purposive sampling strategy to recruit participants. A sample size of 5 participants is required to identify 80% of usability issues, while a sample of up to 15 participants enables identification of 100% of usability concerns [37]. We recruited 10 participants to ensure we identified at least 80% of the usability issues that could arise with the product under investigation. We advertised the study through local pharmacies, researchers’ professional networks, community environments (e.g. grocery stores, community health care centres and libraries), social media on the University of Waterloo School of Pharmacy’s website, Facebook and Twitter page and by approaching previous study participants who had indicated willingness to participate in future studies. Community pharmacists were provided with an approved recruitment script to help them identify potential participants in their practice. If participants were interested in participating, community pharmacist would share their contact information with the research team, after obtaining consent to do so. Since pharmacies were required to dispense medications in the smart blister package, a participant’s community pharmacy had to agree to participate in the study, or the participant had to be willing to transfer their prescriptions to a participating pharmacy. Participants were eligible to take part in the study if they were (1) 18 years of age or older, (2) had more than one chronic disease, (3) on a complex medication regimen (defined as taking five or more oral medications per day, or if taking less than five oral medications per day, taking a more than once-daily dosing schedule for an oral medication), (4) self-managing their medications regularly, (5) able to speak English, and (6) had a cellular phone with SMS messaging capabilities. Participants who were residing in long-term care homes and were on nursing medication administration programs were not eligible to participate due to the potential need to alter their medication management process. Additionally, since smart multi-dose blister package requires users to respond to prompts written in English in order to use the product, individuals who were unable to speak or read English, or individuals with cognitive impairments were excluded due to their inability to respond to prompts adequately.

### Study procedure

**Fig 3:**
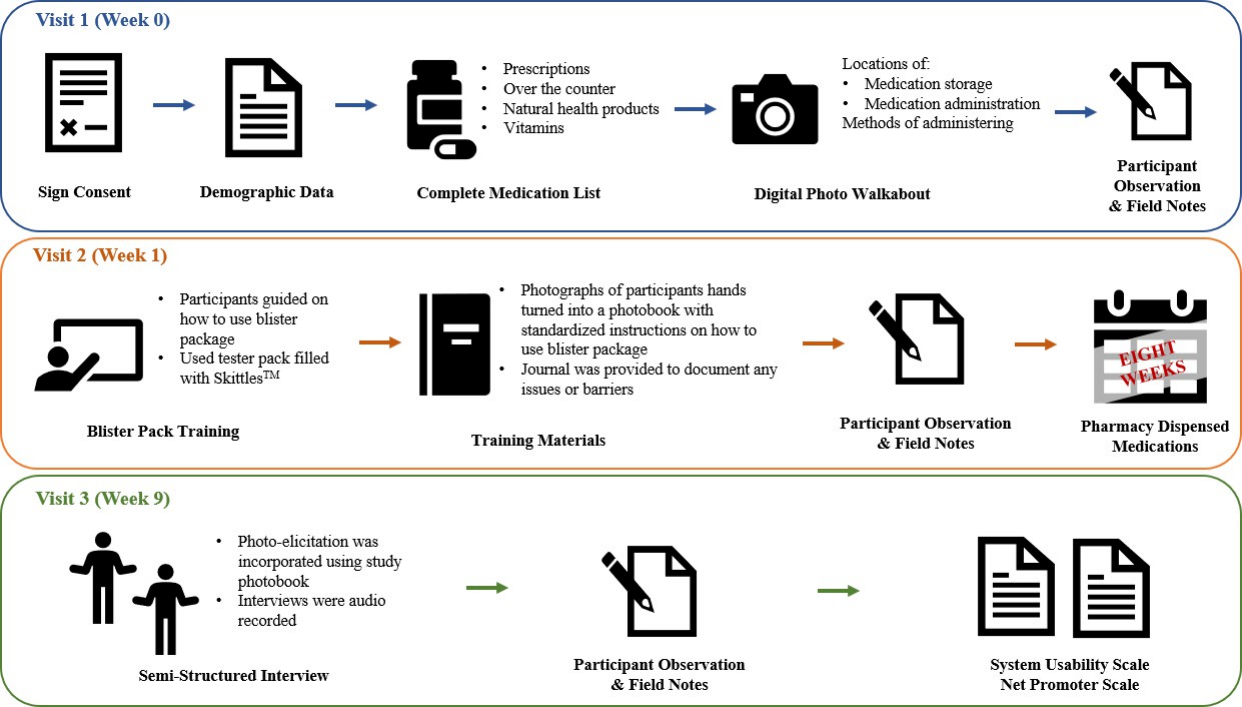
Details of study visits.

Data was collected in 2 large cities in Ontario, from November 2019 to May 2020. Patients were identified by community pharmacies and approached to participate in the study. Participants were asked to complete three in-home patient visits, which ranged from 60 to 90 minutes each. In order to collect data, we used both quantitative and qualitative methods, including in-home participant observations, field notes, digital photo walkabouts (a process of capturing photographs while walking around the place of interest), semi-structured one-on-one interviews using photo-elicitation (a process of utilizing visual methods such as photographs or videos during a participant’s interview), and validated tools such as System Usability Scale (SUS) and Net Promoter Score (NPS) (see Fig 3) [38, 39]. Two of the three researchers (JI, RT and SF) conducted the in-home visits. The coronavirus disease 2019 (COVID-19) pandemic was declared near the end of our study and required us to conduct the last four patient visits virtually.

#### Development of semi-structured interview guide

A semi-structured interview guide was developed by using the constructs from three theoretical frameworks. We utilized constructs from TAM to examine the integration and also incorporated constructs of TPB and COM-B Model to explore an older adult’s medication intake behaviour. We expected that together, the constructs of these theories would reflect the most common determinants of technology use and in-home medication intake behaviour. Any constructs that were overlapping among these frameworks were used once. Additionally, we added questions regarding the concept of integration. We explored integration by incorporating the following three components: usability, functionality and acceptability in the interview guide.

- Usability refers to how specified users can use a product to achieve defined goals [40]. This was assessed by asking older adults if they were able to remove the tablet from the smart blister package.
- Functionality was defined as “the ability of the product to do what it is intended to do” [41]. This was assessed by inquiring questions about the functioning of the alarm and any issues or difficulties related to tablet retrieval from the blister package.
- Acceptability was defined as “acceptance of the product by the end-user” [42]. This was assessed by inquiring about the future intention to use the blister package.

The interview guide was initially developed by two researchers (JI and SF) and further reviewed by two researchers (TP and CM) with research and clinical experience in quantitative and qualitative research, and pharmacy, respectively (see S1 File for a mapping of the interview guide to TAM, TPB, and COM-B). Furthermore, we incorporated the photo-elicitation method during one-on-one interviews. The researcher took multiple photographs of the participant’s hands while they were using the blister package to administer their medications. These photographs were used during the interview process to discuss any issues related to the use of the blister package. Two researchers (JI and SF) conducted one-on-one interviews. The duration of the interview ranged from 45 to 60 minutes. All interviews were audio-recorded. Field notes were written after each interview.

### Data analysis

#### Integration and medication taking behaviour: Semi-structured interview

Each interview was transcribed verbatim by three team members (KP, CH and AP) on Microsoft Word (Microsoft® for Mac version 16.16.13). Two different team members (SF and JI) reviewed the transcripts for accuracy. Four team members (SF, JI, RT and KP) conducted data analysis. These individuals provided a variety of backgrounds including a pharmacist, systems design engineer and health informatics. These complementary backgrounds provided multiple informative perspectives to data analysis. The Qualitative Analysis Guide of Leuven (QUAGOL) framework was used to analyze interview data [43]. Multiple team members (SF, JI, RT and KP) read, reread and analyzed the interviews independently and created a list of concepts to develop the code list. The NVivo 11 (QSR international, Melbourne, Australia) was used to organize and code the data. Following this, two researchers (JI and SF) independently coded the interviews in detail using the code list. Data saturation was reached at the 7th interview, and no new codes were elicited after that. However, we decided to code all interviews to confirm saturation. Researchers (JI and SF) reread the interviews and linked significant passages with the codes on the code list. Following the review, additional codes were added, and codes without links were removed. A codebook was created containing the name of the code, definition and quotes from the interviews. Researchers (SF, JI, RT and KP) had multiple discussions during the process to ensure consistency. Codes were grouped into overarching themes and sub-themes. Themes and sub-themes were mapped back to TAM, TPB and COM-B Model. To ensure the trustworthiness of the data, we performed member checking with all participants. Member checking is a process of participant validation of research findings [44]. All participants were provided with a document outlining the themes and sub-themes of the study in a layman language. Fifty percent of study participants responded to our member checking process and agreed with the interpretation of the results. No changes were made to themes and sub-themes after their feedback.

#### Usability: System Usability Scale

The SUS is a validated subjective assessment used to determine a product’s usability [45]. It consists of 10 statements (five positive and five negative), scored on a five-point Likert scale. Scores range from 0 to 100, where higher scores indicate that a product is more usable [45, 46]. Bangor et al. described scoring systems for the SUS using the following adjectives: *acceptable* (SUS scores above 70), *marginal* (SUS scores between 50–69), and *not acceptable* (SUS scores below 50) [46].

#### Acceptability: Net Promoter Score

The NPS score is a simple tool to assess the overall satisfaction of the user with a product [47]. It consists of a single question with a scale of 0 (very unlikely) to 10 (very likely). The NPS score determines three types of users: (a) Promoters are the users who provided positive comments about the product and respond with a 9 or 10 on NPS scale (b) Passives are the one who are indifferent about the product and respond as 7 or 8, and (3) Detractors are the ones who are not satisfied with the product and answer the NPS question with a 6 or lower. The NPS is calculated by subtracting the percentage of detractors from the percentage of promoters. The score is expressed from -100 to 100 [47]. Positive scores indicate that users are satisfied with the product and would most likely recommend the product [47].

## Results

### Demographics

A total of 26 patients were identified by participating pharmacists. Of the 26, one did not have a cellular telephone, eight refused to participate due to personal reasons, and five did not respond to the researcher’s initial contact. One participant withdrew consent due to ongoing health conditions before the study started. No participants were required to transfer their prescriptions to a participating pharmacy. Ten participants were enrolled, with an average age of 76 years (SD: 11.7, range: 57–88), of whom 80% were female. A total of 70% of participants lived with a spouse or partner, 10% lived with a friend, and 20% lived alone. Participants reported 4.9 medical conditions, on average (SD: 1.6, range: 3–8) (see Table 1).

**Table 1:** Demographic characteristics of participants.

Variable	(N = 10)
**Gender (n, %)**	
** Female**	8 (80.00%)
**Age (years)**	
**Mean ± SD**	76 ± 11.7
**Range**	57–88
**Living Arrangement**	
** Alone**	2 (20.00%)
** Spouse/ partner**	7 (70.00%)
** Others**	1 (10.00%)
**Level of Education**	
** <High School**	3 (30.00%)
** High School**	1 (10.00%)
** College/University**	7 (70.00%)
**Reported Medical conditions**	
** Hypertension**	9 (90.00%)
** Osteoarthritis**	5 (50.00%)
** Mood/anxiety disorders**	5 (50.00%)
** Cancer**	4 (40.00%)
** Ischemic heart disease**	3 (30.00%)
** Asthma/chronic obstructive pulmonary disease**	3 (30.00%)
** Osteoporosis**	3 (30.00%)
** Diabetes**	2 (20.00%)
** Other**	7 (70.00%)
**Number of Medications taken per participant**	
**Mean ± SD**	11.1 ± 5.1
**Range**	5–20
**Rx (mean, ± SD, range)**	7.4 ± 4.7 (4–16)
** OTC/NHP/Vitamins (mean, ± SD, range)**	3.7 ± 1.8 (0–6)
**Medication aids used (n, %)**	
**Yes**	9 (90.00%)
** Pharmacy prepared blister package**	5 (55.55%)
** Patient prepared dossette**	4 (44.44%)
**No**	1 (10.00%)

### Qualitative analysis

Three themes emerged from the qualitative analysis of interviews which are discussed below without any specific hierarchy.

### Themes and sub-themes

#### 1. Factors influencing the medication intake behaviour

*1*.*1*. *Health literacy*. When asked, “why is it important to take medications on time” participants responded in many ways. Some participants had a clear understanding of their medical conditions. They were knowledgeable and aware of the importance of taking medications on time, as prescribed by their healthcare providers.

“I think the ones in the morning and night are more important because they’re the ones [for my] … cholesterol and blood pressure”-011PT

Conversely, some participants did not understand the significance of proper medication intake and lacked the necessary knowledge of dose-time adherence. One participant discussed the importance of taking medications on time in an ambiguous way;

“I think if you can take your medication on time, you make better use of the medication because… as soon as your body… empties, you’re refilling it again and I think that’s a good thing because I think it makes the medication stronger and, better for a person”-012PT

*1*.*2*. *Age-related physical and cognitive changes*. The impact of aging was an emerging sub-theme which came about without a probing interview question. Participants mentioned both physical and cognitive age-related changes while discussing their medication intake routines in their homes. One participant described how, with age, they are experiencing deficits associated with vision and hearing:

“I have a problem with my eye-sight… sometimes … I have to do different things for it. Sometimes I had to get onto the phone and was … having trouble with my ears.” -012PT

Almost all participants mentioned forgetfulness and memory issues due to aging as something that impacted their medication intake.

“As you get older you sometimes think you did something and you turn around [and realize] “oh no I didn’t do it”.”-002PT

*1*.*3*. *Social support system*. Participants described social supports as an important aspect of their in-home medication intake process. Participants mentioned their spouses, children, friends and pharmacy staff as their social support system.

“My son-in-law [is my support system] because he works [at the pharmacy]. He was always the one taking care of [me]. He always brings my pills home for me.”-008PT

*1*.*4*. *Mental and physical workload*. Participants often mentioned that managing multiple medications regularly was a difficult task requiring both cognitive and physical capabilities. One participant discussed the daily cognitive workload involved with their medication intake by using the following quote:

“I’d have pill bottles all over the place… Then [to] try to remember to call the pharmacy to order more, or in when my next delivery was…I try to order the pills on the same day my deliveries come in”-011PT

Another participant discussed how accessing medications from the pharmacy is a difficult task to manage:

“To tell you the truth, it’s [a] pain in the neck. Because, especially in [the] wintertime… I’m able to pick up the medication from [the pharmacy], but you know, with ice and snow…”-014PT

Participants identified that pharmacy prepared blister packages are valuable as they reduce the cognitive and physical workload that is involved in managing complex therapies regularly.

“You gotta take half of this [medication], one or three of this [medication]one or one of this one ugh, it’s much better the way it is in the blister pack”-011-PT

#### 2. Facilitators related to product use

*2*.*1*. *Product simplicity and learnability*. When asked about their experience using the smart blister package, all participants felt it was easy to use.

“I do like that… cause… it was nice and simple to remind you that you did forget which is great”-011PT

Participants found that it was very easy to learn how to use the product and they did not require any ongoing support in terms of the learning process.

“I just uh went ahead and did it so, you, you showed me, you made it quite clear”012-PT

Some of our participants were using regular pharmacy prepared blister package for their medication management and this familiarity with the blister package design also made it easy for them to learn and use the smart blister package.

Some participants perceived that it would be more beneficial if a user starts using the product before they have a memory issue for better learnability.

“I would because I can see the problem getting worse. It’s bound to. You live longer and you get forgetful so it goes with the territory. So, I think it is a good investment with time to learn to use it when you are younger-001PT

*2*.*2*. *User satisfaction*. Participants had mixed reactions with the overall satisfaction with the product use. Some participants were very satisfied with the product, while others expressed that the product would be better if there were some modifications made to the reminder notification system, produce size, and the addition of an audio signal.

“If it was working—, obviously it was to an extent, if everything was the way it is I—would be very comfortable with it”-002-PT

Some participants showed intention to use the smart blister package in the future to manage their medications if needed.

*2*.*3*. *Product induced behaviour change*. Participants stated that using a smart blister package changed their behaviour. They became more aware of taking their medications on time. Some of them mentioned that the reminder function kept them alert.

“It made me more alert to the fact that my medication was waiting for me”-012PT

*2*.*4*. *Familiarity with the technology*. Participants also reported that their understanding of the smart blister package’s technological system was impacted by their prior experience or familiarity with technology. Participants who were using some kind of technology such as a computer, smart TV or tablet understood the product quicker as compared to participants who were not using any technology-based devices in their daily routines.

“I didn’t mind [using the smart blister pack] because I am used to … technology for the most part”-009PT

*2*.*5*. *Feedback from social circle*. Another important factor that emerged during the interview analysis was the feedback from the different social circles. Participants reported their spouses or children felt less worried about them as the smart blister package helped manage their medications in a safe and organized manner.

“I think they will probably be more… satisfied that I that I won’t mess up my prescriptions. I do get forgetful and I do get mixed up and sometime I take the medication and then I can’t remember if I’ve taken it and with that I didn’t have any question as to whether I took it or no”-009PT

Only one participant mentioned their paid caregiver did not feel the smart blister package was beneficial due to the participant’s inexperience and difficulty retrieving tablets appropriately. However, many participants did not care about what other people thought of them while using the device.

“I don’t care what other people think of me”-014PT

Participants also mentioned that the use of the device promoted a positive interaction with their pharmacist.

*2*.*6*. *Product induced positive emotional response*. Emotional responses such as a sense of relief, feeling of safety and less worry were reported frequently by most participants.

“Well there again I thought it was good because [….] it is just [like] a little bit of a secure, a security blanket”-002-PT

*2*.*7*. *Perceived usefulness in other patient populations*. All participants showed interest in using the product in the future if they required any assistance with their medication management. Most participants perceived the product’s reminder function as most useful for people suffering memory issues and those who forget to administer their medications due to other reasons.

“I don’t need that now (ok). But if I had losing my memory or you know when you get older …. I would”- 013-PT

Participants also discussed that one of the potential users of this type of product is nursing home patients. One of the participants quoted;

“I really think that that would be very useful to be used in nursing homes, for nurses that give out medications. They would probably appreciate the ease of use and the reminder for them”-009-PT

#### 3. Barriers to product use

*3*.*1*. *Product design*. Participants mentioned product features such as device size, ability to lock and portability as factors to consider when incorporating the smart blister package for in-home medication management. Almost all participants said that the device’s size affected their ability to store it in the same place where they previously kept their medications or adherence aid.

“[The blister pack] was too large to-to go where I… normally put my other one”-015PT

Participants also mentioned that the ability to transport the smart blister package was an important aspect for them.

“When I first started getting ready to book my trip, I was concerned about whether or not I could travel with them on the airplane”- 009PT

During the interview discussion, a participant mentioned the smart blister package did not have a locking feature or notification function if you open the wrong blister cavity.

“I know that [I made] a mistake but some people, you know…they open it and they go there are my pills and then they take… the wrong one at the wrong time”-014-PT

Participants also reported that while they were opening the blister cavity, tablets would fall out from their hands.

“The only thing is when I was pushing the bubble down to get my pills out, of the package I would always lose it”-012PT

*3*.*2*. *Product inconsistency*. Participants found that the messaging system was inconsistent. There were a few instances where participants received reminder messages to administer their medications when they had already taken them or did not receive the notifications even though the administration time had passed.

“I would not, … I would not rate the system as being reliable.”-016PT

*3*.*3*. *Technology access*. The smart blister package required a cell phone that could receive text messages to demonstrate its full functionality. Some participants identified that the availability of the necessary technology to use the smart blister package is of significant importance and can impact its integration for in-home medication management.

“Well if my cell phone isn’t working. My husband has a regular little phone but it [cannot receive text messages]. I don’t think you can hook up with that”-001-PT

*3*.*4*. *Financial concerns*. Cost was also reported as an essential factor affecting the use of adherence technology. Some participants showed a willingness to pay for these technologies if they needed a reminder for their medication intake. However, some participants did not agree with paying for these technologies if their provincial or private health insurance did not cover them.

“But there is a financial thing involved too […] you know? And a lot of people… older people… they [are] living on a tight budget… and the pension [is] no[t] high [….] And that is an extra burden financial for people”- 014PT

*3*.*5*. *Product induced negative emotional response*. Some participants felt panic and frustrated when retrieving the tablets from the blister package. Most of these feelings were reported earlier in the study due to participants being unfamiliar with the system and having difficulty in tablet retrieval.

“I get very frustrated. I get frustrated if I try different methods of how to get [the tablets] out”-008PT

Some participants felt worried as they did not fully understand how the reminder function worked and who was sending them the reminder messages. For instance, one participant called their pharmacy a few times to inquire about why she is not receiving messages. The community pharmacist then ensured her that since she took her medications before her scheduled time the system did not generate any reminder messages for her.

Some of the participants felt that due to the product use they had lost their autonomy, which was concerning for them.

“It’s just that I wouldn’t wanna have to rely on somebody”-015-PT

*3*.*6*. *User’s physical and cognitive abilities*. Participants reported that utilizing the smart blister package required particular physical and cognitive abilities. Some participants felt that retrieval of the tablets was challenging or not possible for patients with certain medical conditions such as arthritis or Parkinson’s disease.

“Like somebody that was really old and… if their fingers were very… arthritic or something… I think an older… senior would have a terrible time with that”-008PT

Participants also reported that people needed to have some cognitive capacity to understand and use the technology effectively. They expressed that age may impact these cognitive abilities and, ultimately, the use of the product.

“The situation would be though that if I were to move to using a blister pack…. it would be because my mental capability had decreased and that in itself would likely decrease my capability of using… technical…. services… if I had to use a blister pack… it would only be because I would have deteriorated and by the deterioration, I would not be able to use…. cell phones… probably”-016PT

### Findings mapped to theoretical frameworks

**Fig 4:**
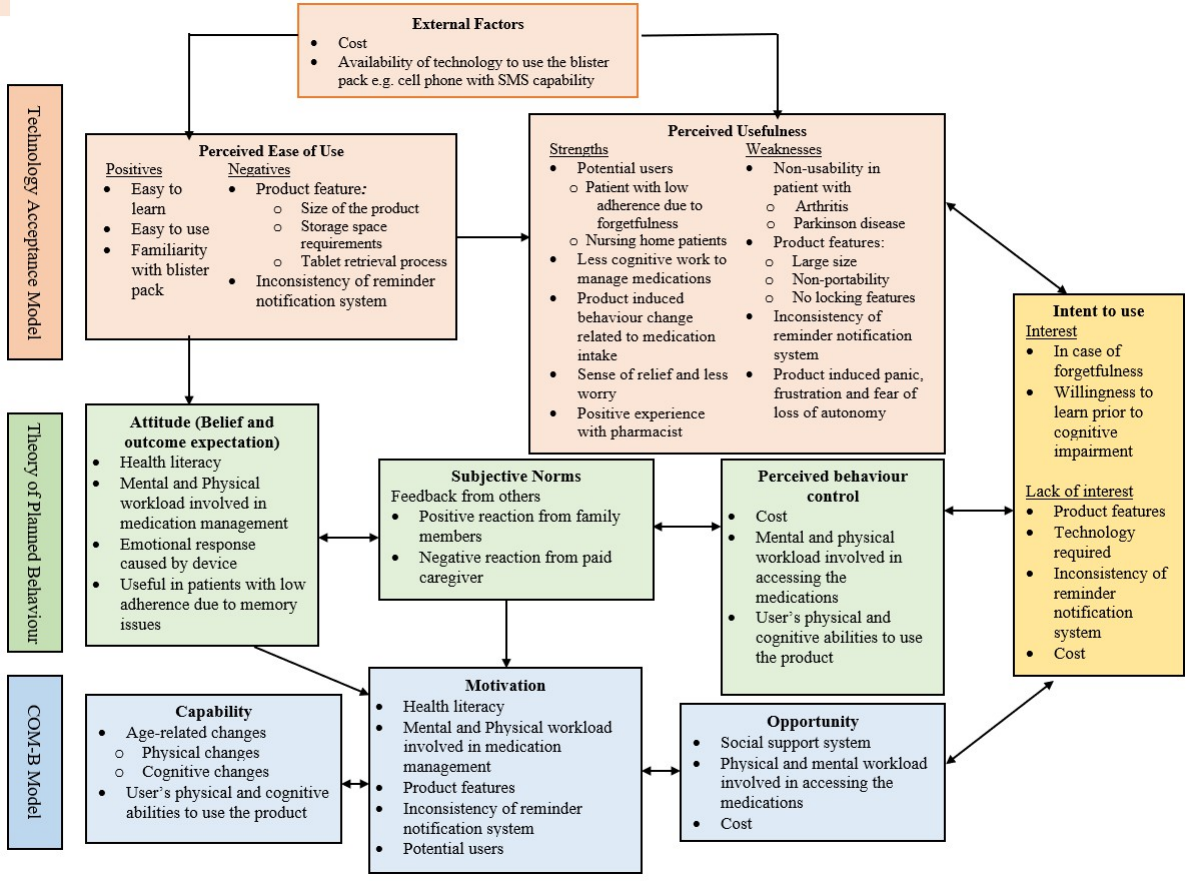
Factors impacting integration of smart packaging.

The themes and sub-themes identified by the qualitative analysis of interviews were mapped to TAM, TPB and COM-B Model (see Fig 4).

### Quantitative analysis

#### Usability (SUS)

The mean SUS score for the blister package was reported to be 75.50 (range: 37.5–92.50).

#### Acceptability (NPS)

Of the 10 participants, only eight completed the NPS score, of which 37.5% (N = 3) were detractors, 37.5% (N = 3) were promoters and 25% (N = 2) were passive. The overall NPS score was found to be 0.

## Discussion

### Principal findings

The results of this study identified numerous factors affecting the medication intake behaviour as well as barriers and facilitators in using a smart adherence product for in-home medication management. To the best of our knowledge, this is the first study to use an integrated theoretical framework based on TAM, TPB and COM-B Model to investigate the integration of a smart adherence product by exploring the challenges and facilitators to use the product, and outline medication intake behaviour in older adults with chronic diseases.

### Medication intake behaviour

The use of TPB and COM-B model further confirmed that an older adult’s medication intake behaviour depends on multiple factors. These findings further add to the existing literature focused on factors affecting the medication adherence [10]. Our study participants reported health literacy, social supports, age-related changes, and mental and physical workload involved in managing complex prescriptions as important determinants to impact medication intake behaviour.

Health literacy can be defined as "the degree to which individuals have the capacity to obtain, process, and understand basic health information and services needed to make appropriate health decisions" [48]. For medication intake specifically, health literacy not only involves the ability to understand prescription information, but also entails a patient’s knowledge of the prescribed drug related to the medical condition it’s being used for. Our study results indicated that some older adults were very well informed about medication intake while some lacked this understanding. This lack of understanding has been linked as an important patient related determinant of non-adherence [10, 49]. A recent meta-analysis of the role of health literacy in diabetes knowledge, self-care and glycemic control showed that health literacy plays a significant role in disease management [50].

Besides health literacy, social support was identified as an important factor affecting the in-home medication intake process. We defined social support systems as any individual involved in the medication management process. Studies have shown that social support systems can play an essential role in chronic disease management and improve quality of life by providing patients with helpful resources [51, 52]. The availability of social supports has been positively co-related with medication adherence in various chronic conditions [52–54]. Although all of our participants were independently managing their medications, they still identified their spouses, children, friends and even pharmacy staff as their social support system. These people were involved in the participant’s medication management process in various ways ranging from picking up medications from the pharmacy to reminding the participant to take their medications. Participants living alone indicated their community pharmacist as a helpful resource.

People with chronic diseases are often managing complex therapies on a regular basis, requiring both cognitive and physical abilities of the patient [10, 13]. In older patients, age-related physical and cognitive changes may impact the medication management and intake process. Our participants indicated age-related forgetfulness as an important change affecting their ability to manage medications. Physical deficits such as hearing loss and vision impairment can affect the ability to read prescription information from the label or hear directions on how to appropriately use medications [55]. Managing multiple therapies requires one’s ability to not only remember to take medications, but to also have a system in place for ordering medications from the pharmacy on time [10, 56]. Therefore, the clinician should always determine a patient’s physical and mental capacity along with other determinants before initiating a complex medication plan to ensure adherence.

### Facilitators and barriers

The TAM framework along with TPB and COM-B model assisted us to outline certain facilitators and barriers related to integrating a smart adherence product for in-home patient use.

#### Facilitators

Two facilitators that were identified in this study include ease of use and ease of learning how to use of the product. Both of these facilitators impact a person’s decision to use the technology regularly. Although most of the study participants did not feel they needed the to use the product at this current time, they all identified the perceived usefulness of this product for patients with memory impairment or unintentional non-adherence due to forgetfulness. Additionally, participants showed their intention to use the product in the future if needed.

Another facilitator identified was the prior exposure or familiarity with the product technology. Participants who had familiarity with the technology embraced the smart blister package quickly and felt comfortable using it. We believe that pre-existing familiarity with technology increased older adults’ confidence in using the smart blister package during this study.

Positive feedback from non-participants was identified as another facilitator. Participants reported that their family members’ positive responses made them feel motivated to use the smart blister package and using the blister package provided a sense of relief for their family members. Involving family members, if possible, when recommending these technologies should be considered to ensure continuous use. Moreover, participants felt that the product use improved their interaction with their healthcare providers.

Positive emotions such as experiencing a sense of relief and less worry due to the use of the product was another facilitator found in this study impacting product integration. A study discussing user’s perspectives on adherence products has cited similar results. The study reported that patients prefer to use an adherence product for their medication management if it provides them with a feeling of less worry and sense of assurance that they did not miss any doses [57].

Our analysis also indicated that product-driven behaviour change occurred due to the smart blister package’s reminder notification function. Most of our study participants felt obligated to take their medications on time as they perceived that someone was investing time and effort to take care of them. This change in behaviour can be very helpful in addressing unintentional non-adherence due to forgetfulness. However, due to the duration of the study, we are unable to comment on if this behaviour change will be sustained over time. A systematic review of patient reminder systems indicated the importance of measuring sustainability of behaviour change as these patient reminder systems can be recommended as both long and short term solutions to help initiate behaviour change [58]. We recommend future studies assess sustainability of this behaviour change as this will help inform how we should be recommending these tools to patients.

#### Barriers

Product characteristics such as the large size, limited portability and lack of safety features were identified as barriers impacting the smart blister package’s regular use. Additionally, participants found specific issues related to tablet retrieval from the blister package or inconsistencies in the reminder notification system. Previous studies have reported reliability of the technology as a common concern related to technology use in older adults[19, 59, 60]. For example, a previous study based on older adults’ perception about various technologies used in places like home, work and healthcare, reported that participants disliked products that do not function in a reliable manner [60]. A literature review based on daily activity monitoring technologies such as personal alarms, fall detection devices, wearable devices, etc. for older adults discussed non-reliability of the devices as one of the challenges to implement these technologies [61]. Patients and family members often use these products to experience a sense of relief or less worry regarding the medication management process. Therefore, the product’s non-reliability should be addressed as it may very well impact the long-term use of these products.

In order to use the smart blister package, participants needed to have a cellular telephone with the capability of receiving SMS messages. Recruitment of older adults in this study was challenging as many continue to use landline telephones and do not have cellular phones. A 2019 Canadian report on the use of smart technology by Canadian seniors aged 73 years and older has shown that 39% of seniors have no cellular phone at all, 27% own a basic cell phone, and 34% own a smartphone [62]. Some older adults shared one cellular phone between both partners; this would produce a challenge when sending notifications for medication doses. The sharing of a cell phone was a surprising finding as most of the literature suggests that technology use is increasing in older adults. The use of smart phones in Canadian adults aged 25 to 44 years and 45 to 64 years of age has been reported as 97.1% and 87%, respectively [63]. Therefore, the future generation of older adults will likely not face the same challenge as they may be more familiar and well versed with technology use. However, the landscape of medication adherence technologies may also change overtime.

Cost was considered another important barrier to the use of such products. A recent review reported that the cost associated with adherence technologies could vary from a few dollars to a few hundred dollars [23]. Also, some of these products have a cost associated with data charges and connectivity fees. Studies have reported that the inability to pay for medications negatively impacts long-term therapy adherence [64, 65]. Generally, older adults are on a fixed income and cannot afford medications or other health-related devices if public or private insurance plans do not cover them. Therefore, financial implications should be considered when offering these technologies.

The negative emotional responses caused by product use was another critical barrier to consider. Participants reported a range of emotions while using the smart blister package. Although the technology provided them a sense of relief and less worry, they did experience the emotions of being panicked and frustrated at some occasions, more specifically at the start of the study. Studies have reported that older adults like to adopt technologies; however, they perceive themselves as less confident and self-sufficient to use new technology [66]. The lack of confidence can create emotional responses of frustration and panic. However, in our study some participants found the use of the smart blister package became easier over time, thus improving their confidence to use the product. These emotional experiences caused by a product may impact decision making around its continuous use.

Certain medical condition such as arthritis or Parkinson’s disease can impact an older adult’s ability to retrieve tablets from different packaging [55, 67, 68]. Additionally, patients with compromised cognitive functioning, may face challenges using the blister package due to a limited understanding the system [69, 70]. Both age-related and disease-related decline in sensory and cognitive functioning may significantly impact a person’s ability to use the technology. Patients with hearing loss may not hear the sound of a text message if it is not in close proximity. Similarly, patients with cognitive impairment may not remember how to respond or react to the reminder function.

### Usability and acceptability

The SUS is a popular subjective assessment used to determine the usability of a product [45]. For example, the mean SUS score for microwave is reported to be 86.9, for Microsoft Word ® is 76.2 and for using an ATM is 81.8 [71]. Some research has been conducted in assessing the usability of electronic medication adherence products such as the smart multidose blister package tested in this study, however, there is no benchmark for SUS scores of such products. One usability and workload study determined the mean SUS score for 21 electronic products was 52.28 (SD: 28.52; range: 0–100) [72]. Our study reported a higher mean SUS score for the smart multidose blister package. As per Bangor et al. ‘s acceptability scale, this product is acceptable to use [46].

The NPS score is used to evaluate the overall satisfaction of the user about the product [47]. The NPS score was reported as 0. The score indicated that the smart adherence product had an equal number of detractors and promoters. Participants who were detractors on the NPS scale reported that they would recommend the product if its design and reliability were improved.

### Strengths and limitations

The most significant strength of this study is the methodology used. Use of ethnography-based data collection methods has provided detailed, comprehensive and in-depth information about the complexities related to in-home medication intake behaviours. To the best of our knowledge, this is the first study that used an ethnographic data collection methodology to understand medication intake behaviours and examine the integration of a smart adherence technology for in-home use in older adults with chronic diseases by utilizing a combination of TAM framework along with two health behaviour theories, e.g. TPB and COM-B Model. It has been argued that TAM does not adequately address the acceptance of health-related technology, and certain other factors can influence the incorporation of technologies into daily patient use; therefore, two health behaviour theories were used to identify additional factors. Additionally, the photo-elicitation method was used during the one-on-one interviews to enhance participation and gather richer data. Furthermore, the use of the team-based approach with complementary backgrounds in pharmacy, systems design engineering and health informatics to conduct the data analysis provided interprofessional triangulation and added rigour to the study. In addition to strengths, this study has limitations such as brief duration of in-home observations and the change of interviewing atmosphere from in-person to over-the-phone due to COVID-19.

## Conclusion

This study’s findings support existing literature and further document barrier and facilitator determinants which can be incorporated into adherence technologies for in-home patient use. The integrated use of TAM, TBP, and COM-B Model, highlighted how the identified barriers and facilitators in this study are interconnected and can impact an older adult’s intention to incorporate such technology-based products into their daily medication intake routine. Our study results indicate that the smart adherence technology was easy to use, acceptable by older adults and can be a useful tool for in-home medication management. However, particular areas of improvement regarding product design and reliability should be considered. These findings provide an opportunity for industry partners to improve product design and reliability in a real-world context. Moreover, future studies should be planned to assess the healthcare outcomes, cost saving and sustainable product driven behaviour change by implementing these technologies in older adults who are at high risk of non-adherence. A study with this focus may lead to a discussion with policymakers to identify new cost models that promote affordable access to these technologies.

## Supporting information

S1 File Interview guide. (DOCX)

S2 File COREQ checklist. (PDF)
