# Supplementary figures and images for: Integration of a smart multidose blister package for medication intake: A mixed method ethnographic informed study of older adults with chronic diseases
Source: PLoS One. 2022 Jan 21;17(1):e0262012. doi: 10.1371/journal.pone.0262012 (PMC8782488; doi:10.1371/journal.pone.0262012)

# **Appendix A: Interview Guide**


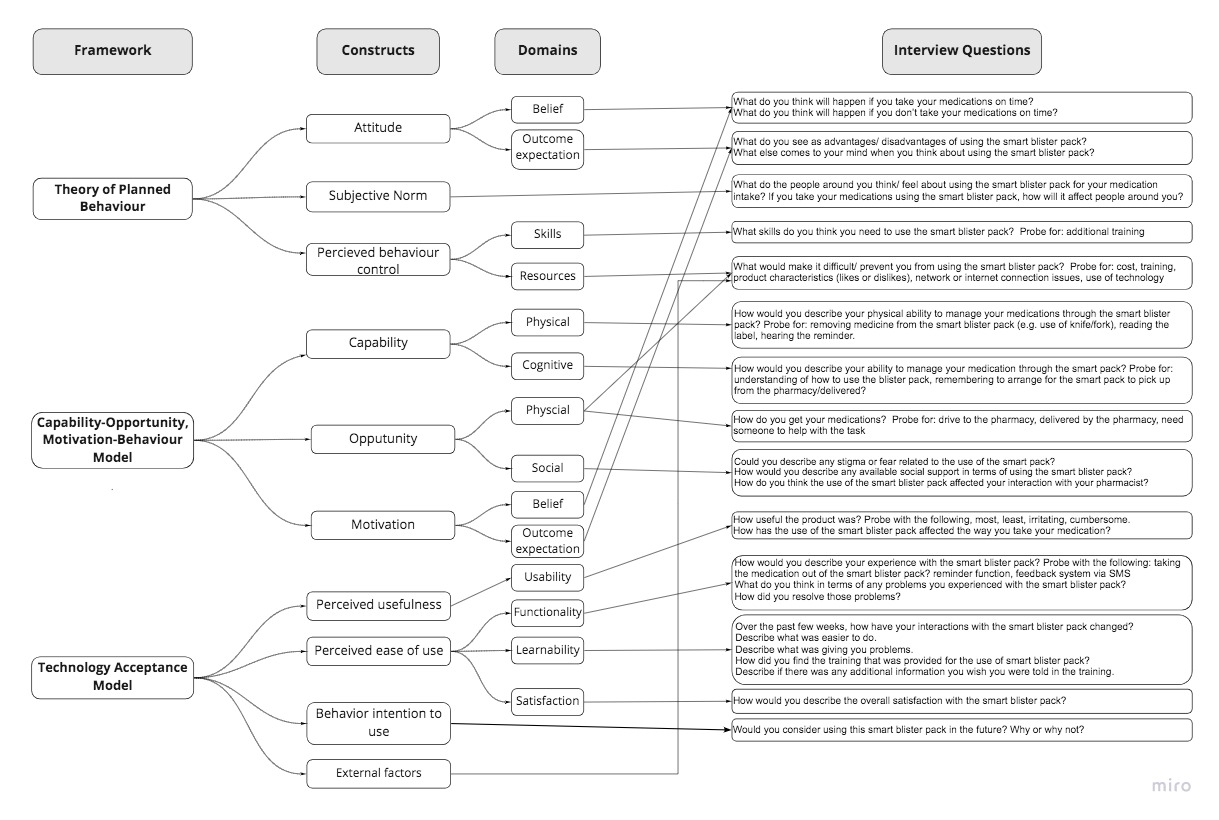

Supplement: S1 File — (DOCX) [file pone.0262012.s001.docx]
